# Enhanced Nitrite-Mediated Relaxation of Placental Blood Vessels Exposed to Hypoxia Is Preserved in Pregnancies Complicated by Fetal Growth Restriction

**DOI:** 10.3390/ijms22094500

**Published:** 2021-04-26

**Authors:** Teresa Tropea, Carina Nihlen, Eddie Weitzberg, Jon O. Lundberg, Mark Wareing, Susan L. Greenwood, Colin P. Sibley, Elizabeth C. Cottrell

**Affiliations:** 1 Maternal and Fetal Health Research Centre, Division of Developmental Biology and Medicine, Faculty of Biology, Medicine and Health, University of Manchester, Manchester M13 9WL, UK; mark.wareing@nhs.net (M.W.); susan.l.greenwood@manchester.ac.uk (S.L.G.); colin.sibley@manchester.ac.uk (C.P.S.); 2 Manchester Academic Health Science Centre, Manchester University NHS Foundation Trust, St. Mary’s Hospital, Manchester M13 9WL, UK; 3 Department of Physiology and Pharmacology, Karolinska Institute, SE-171 77 Stockholm, Sweden; carina.nihlen@ki.se (C.N.); eddie.weitzberg@ki.se (E.W.); jon.lundberg@ki.se (J.O.L.)

**Keywords:** pregnancy, fetal growth restriction, placental insufficiency, placenta, chorionic plate vessels, nitric oxide, nitrite, vasorelaxation

## Abstract

Nitric oxide (NO) is essential in the control of fetoplacental vascular tone, maintaining a high flow−low resistance circulation that favors oxygen and nutrient delivery to the fetus. Reduced fetoplacental blood flow is associated with pregnancy complications and is one of the major causes of fetal growth restriction (FGR). The reduction of dietary nitrate to nitrite and subsequently NO may provide an alternative source of NO in vivo. We have previously shown that nitrite induces vasorelaxation in placental blood vessels from normal pregnancies, and that this effect is enhanced under conditions of hypoxia. Herein, we aimed to determine whether nitrite could also act as a vasodilator in FGR. Using wire myography, vasorelaxant effects of nitrite were assessed on pre-constricted chorionic plate arteries (CPAs) and veins (CPVs) from normal and FGR pregnancies under normoxic and hypoxic conditions. Responses to the NO donor, sodium nitroprusside (SNP), were assessed in parallel. Nitrate and nitrite concentrations were measured in fetal plasma. Hypoxia significantly enhanced vasorelaxation to nitrite in FGR CPAs (*p* < 0.001), and in both normal (*p* < 0.001) and FGR (*p* < 0.01) CPVs. Vasorelaxation to SNP was also potentiated by hypoxia in both normal (*p* < 0.0001) and FGR (*p* < 0.01) CPVs. However, compared to vessels from normal pregnancies, CPVs from FGR pregnancies showed significantly lower reactivity to SNP (*p* < 0.01). Fetal plasma concentrations of nitrate and nitrite were not different between normal and FGR pregnancies. Together, these data show that nitrite-mediated vasorelaxation is preserved in FGR, suggesting that interventions targeting this pathway have the potential to improve fetoplacental blood flow in FGR pregnancies.

## 1. Introduction

Fetal growth restriction (FGR) is the failure of a fetus to achieve its biological growth potential in utero and affects up to 5–10% of pregnancies [1]. This pregnancy complication is a clinically important problem, both perinatally and into adult life. In the short term, low birth weight increases the risk of perinatal mortality and morbidity [2] and contributes significantly to a higher incidence of stillbirth [3]. In the long term, FGR is associated with an increased risk of metabolic syndrome [4], cardiovascular pathologies [5] and impaired neurological and cognitive development [6] in adult life.

In the absence of maternal factors (hypertension, preeclampsia, malnutrition, smoking) and fetal factors (aneuploidy, malformations, infections), FGR most commonly results from placental insufficiency, in which reduced uteroplacental circulation compromises the delivery of oxygen and nutrients to the fetus [7]. In a normal pregnancy, gestational increases in maternal blood volume together with decreased vascular resistance allow the delivery of sufficient blood flow to the placenta, and the uteroplacental vasculature undergoes extensive remodeling to allow adequate fetal growth and development [7]. In pregnancies complicated by placental insufficiency, uteroplacental vascular resistance is high and Doppler flow-velocity waveforms show a high pulsatility index and absent or reversed diastolic blood flow in the umbilical circulation, which indicate impaired placental perfusion and correlate with FGR [8].

As the placenta is not innervated [9], vascular resistance is completely dependent on circulating factors, amongst which nitric oxide (NO) is the key physiological vasodilator of the fetoplacental vasculature [10]. By controlling blood flow to the placenta and regulating fetoplacental vascular resistance [10,11], NO signalling is critical for the maintenance of adequate blood supply to the fetus during pregnancy. Evidence suggests that reduced NO production and/or bioavailability is associated with FGR [12,13] and clinical studies investigating the use of NO-donors in vascular complications of pregnancy aim to enhance fetoplacental blood flow [14].

In addition to endogenous production, via the nitric oxide synthase (NOS) enzymes, dietary nitrate provides a source of NO-generating potential through sequential reduction of nitrate to nitrite, and subsequently to NO, particularly when oxygenation is reduced [15]. We have previously demonstrated significant vasorelaxation to nitrite in human chorionic plate vessels from normal pregnancies, an effect that was enhanced under conditions of hypoxia used to mimic the reduced oxygenation associated with impaired fetoplacental blood flow [16]. Here, we investigated whether placental vessels from FGR pregnancies also demonstrate vasorelaxation to nitrite and whether this response was still enhanced under hypoxic conditions. In addition, we determined the concentrations of nitrate and nitrite, as independent anions, in the fetoplacental circulation of normal and FGR pregnancies.

## 2. Results

### 2.1. Nitrite-Mediated Vasorelaxation of Human Chorionic Plate Vessels Is Enhanced by Hypoxia in Both Normal and FGR Pregnancies

We assessed the reactivity to nitrite of CPAs and CPVs isolated from both normal and FGR pregnancies. The demographic and clinical details of the placenta donors are summarised in Table 1.

**Figure 1:**
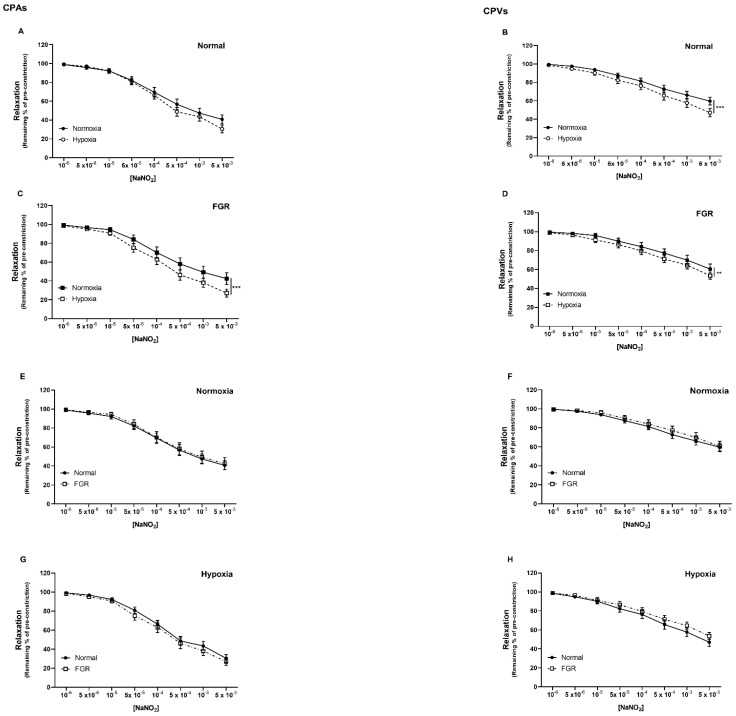
Nitrite−mediated relaxation of human chorionic plate vessels is enhanced by hypoxia in both normal and FGR pregnancies. Concentration−dependent vasorelaxant effect of NaNO_2_ on: normal CPAs (**A**) and CPVs (**B**), FGR CPAs (**C**) and CPVs (**D**), in normoxia and hypoxia; normoxic CPAs (**E**) and CPVs (**F**), hypoxic CPAs (**G**) and CPVs (**H**), from normal and FGR pregnancies. ** *p* < 0.01, *** *p* < 0.001, normoxia vs. hypoxia. *n* = 15–32 placentas per group.

Sodium nitrite (NaNO_2_, 10^−6^–5 × 10^−3^ M) caused a concentration-dependent vasorelaxation in CPAs and CPVs, from both normal and FGR pregnancies (Figure 1).

In vessels from normal pregnancies, hypoxia did not affect reactivity in CPAs (Figure 1A), but significantly enhanced relaxation to nitrite in CPVs (*p* < 0.001; Figure 1B). In vessels from FGR pregnancies, hypoxia significantly enhanced the NaNO_2_-dependent relaxation in both CPAs (*p* < 0.001; Figure 1C) and CPVs (*p* < 0.01; Figure 1D).

Vasorelaxation in response to NaNO_2_ was not different between normal and FGR CPAs and CPVs, under either normoxia (Figure 1E,F) or hypoxia (Figure 1G,H). Maximal relaxation to NaNO_2_ (Vmax) was enhanced in conditions of hypoxia (*p* < 0.05; Supplementary Table S1), whereas sensitivity to NaNO_2_ (LogEC_50_) was not different.

### 2.2. Sodium Nitroprusside-Mediated Relaxation Is Enhanced by Hypoxia in Human Chorionic Plate Veins from Both Normal and FGR Pregnancies

**Figure 2:**
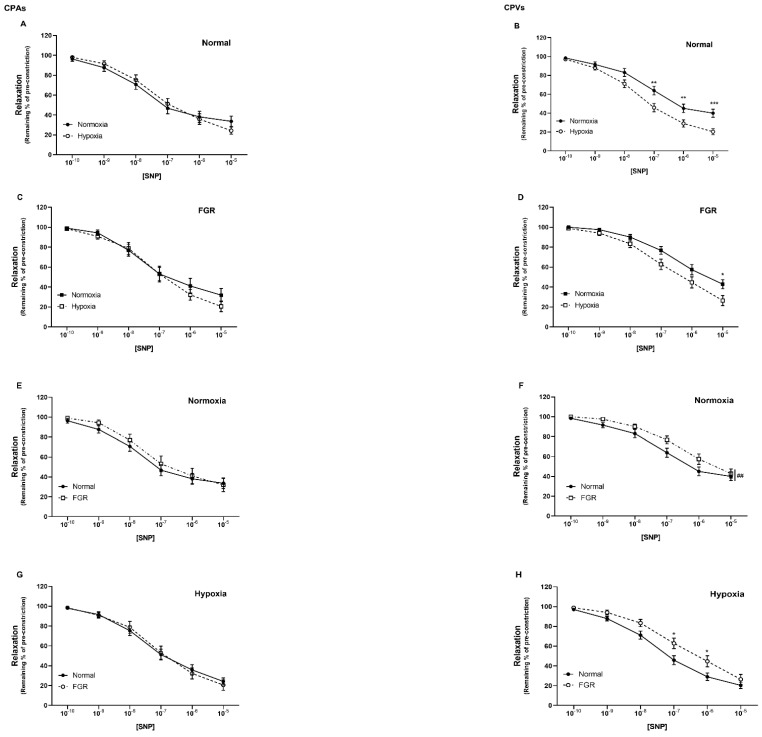
Sodium nitroprusside−mediated relaxation is enhanced by hypoxia in human chorionic plate veins from both normal and FGR pregnancies. Concentration−dependent vasorelaxant effect of SNP on: normal CPAs (**A**) and CPVs (**B**), FGR CPAs (**C**) and CPVs (**D**), in normoxia and hypoxia; normoxic CPAs (**E**) and CPVs (**F**), hypoxic CPAs (**G**) and CPVs (**H**), from normal and FGR pregnancies. * *p* < 0.05, ** *p* < 0.01, *** *p* < 0.001, normoxia vs. hypoxia; ## *p* < 0.01, normal vs. FGR. *n* = 14–30 placentas per group.

The NO donor sodium nitroprusside (SNP; 10^−10^–10^−5^ M) induced dose-dependent relaxation in CPAs and CPVs from both normal and FGR pregnancies (Figure 2).

In pre-constricted CPAs from normal pregnancies, vasorelaxation to SNP was not different between normoxic and hypoxic conditions (Figure 2A), but was significantly enhanced in CPVs under hypoxia (*p* < 0.0001; Figure 2B). Similarly, in vessels from FGR pregnancies, SNP induced greater relaxation in CPVs under conditions of hypoxia (*p* < 0.05; Figure 2D), whilst there was no effect of oxygen tension in CPAs (Figure 2C).

Under conditions of either normoxia or hypoxia, respectively, comparison between normal and FGR vessels showed no difference in CPAs (Figure 2E,G), whereas there was a significant reduction of vasorelaxation in response to SNP in CPVs from FGR pregnancies (*p* < 0.01, *p* < 0.0001; Figure 2F,H, respectively).

Maximal relaxation to SNP (Vmax) was significantly increased under conditions of hypoxia (*p* < 0.05; Supplementary Table S1), whereas sensitivity to SNP (LogEC_50_) was not significantly altered. In terms of differences between vessels from normal and FGR pregnancies, sensitivity but not maximal relaxation to SNP was significantly reduced in FGR CPVs compared to normal vessels (*p* < 0.05; Supplementary Table S1).

The enhanced relaxation to both NaNO_2_ and SNP under conditions of hypoxia was not due to differences in the pre-constriction responses to the thromboxane mimetic (Supplementary Table S2); hypoxia did not alter U46619 dose-responses in CPAs or CPVs from either normal of FGR pregnancies (Supplementary Figure S1). However, there were significant differences in U46619 dose-responses between the different vessel types and between pregnancy groups. Constriction to U46619 was significantly greater in CPAs compared with CPVs (*p* < 0.0001; Supplementary Figure S1 and Table S2). In addition, placental blood vessels from FGR pregnancies exhibited differential reactivity to U46619, with enhanced constriction in CPAs and attenuated constriction in CPVs (*p* < 0.05 and *p* < 0.01, respectively; Supplementary Figure S1 and Table S2).

### 2.3. Fetal Plasma Concentrations of Nitrate and Nitrite Are Not Different between Normal and FGR Pregnancies

**Figure 3:**
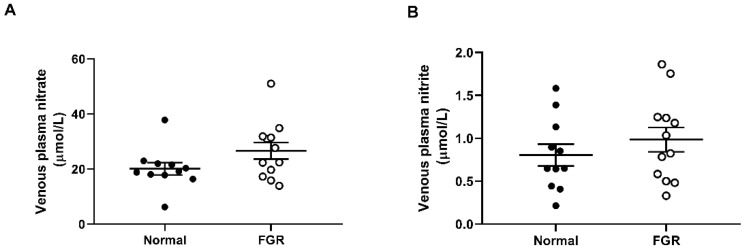
Fetal plasma concentrations of nitrate and nitrate are not different between normal and FGR pregnancies. Nitrate (**A**) and nitrite (**B**) concentrations (µmol/L) measured in umbilical vein plasma from both normal and FGR pregnancies. *n* = 11–12 per group.

Concentrations of nitrate and nitrite were determined in umbilical vein plasma samples from both normal and FGR pregnancies. In normal and FGR samples, respectively, concentrations of nitrate were 20.1 ± 2.2 and 26.6 ± 3.0 µmol/L (Figure 3A) and concentrations of nitrite were 0.8 ± 0.1 and 1.0 ± 0.1 µmol/L (Figure 3B). There was no significant difference between normal and FGR pregnancies in the concentration of either anion.

## 3. Discussion

The results of this study demonstrate that there is similar nitrite-dependent vasorelaxation in human chorionic plate vessels from pregnancies complicated by FGR as compared with vessels from normal pregnancies. The only exception was the enhanced nitrite-mediated vasorelaxation under conditions of hypoxia of CPAs from FGR pregnancies; this oxygen-dependent enhancement in nitrite responsiveness was not seen in CPAs from normal pregnancies. It is possible that this reflects alterations in the mechanisms underlying nitrite reduction to NO within the arterial vessel wall following prolonged exposure to a lowered oxygen tension, as is predicted to occur in FGR [17].

The present findings also confirm and extend our previous observations in normal pregnancy [16] showing vasorelaxant responses to nitrite to be higher in CPAs than CPVs; this same pattern is observed in FGR. We have previously speculated that the lower partial pressure of oxygen in CPAs [18] may increase the sensitivity to nitrite in the placental arterial vascular bed, compared with the venous circulation; alternatively, it may reflect differences in vascular wall structure [16]. Mechanisms underlying nitrite-dependent vasorelaxation in the placental vasculature remain unknown. In our previous study [16], we have demonstrated that known nitrite reductases such as xanthine oxidoreductase, mitochondrial aldehyde dehydrogenase, mitochondrial bc1 complex and NOS enzymes were not involved in the vasorelaxation of human chorionic plate vessels to nitrite. The complete abolition of nitrite-mediated vasorelaxation in the presence of the soluble guanylate cyclase (sGC) inhibitor ODQ not only demonstrated the involvement of the downstream sGC signalling pathway, but we speculate that these data could also suggest the potential contribution of heme-containing globin family proteins in mediating vasorelaxation in response to nitrite [16].

Similarly to the observed nitrite-mediated effects, hypoxia increased vasorelaxation to the exogenous NO donor, SNP, significantly in CPVs but not in CPAs. Comparison between normal and FGR vessels in response to SNP under conditions mimicking physiological oxygen tension showed no difference in CPA reactivity. These data are in contrast with previous work demonstrating significantly greater relaxation to SNP in CPAs from FGR, compared with normal pregnancies [19]. The major difference with the present study is the dose-range of SNP tested; the highest concentration used here was 10 times lower than in the previous study [19], which may explain the lack of difference in our experimental groups. Interestingly, we found that vasorelaxation to SNP was significantly reduced in CPVs obtained from FGR as compared with normal pregnancies. This reduced potency of SNP to relax placental veins in FGR is notable, especially given that nitrite responsiveness was not different between CPVs from normal and FGR pregnancies. However, differences in gestational age between the two groups represent a limitation of the current study. Not unexpectedly, pregnant women with FGR delivered significantly earlier than normal women. Studies performed in placental veins obtained from preeclamptic pregnancies demonstrated that sensitivity to SNP was greater in full term compared to premature placentas, regardless of pathological state, and was similar between preeclamptic and normotensive pregnancies at similar gestational ages [20].

The overall enhancement of hypoxia-mediated vasorelaxation has been proposed as a key regulator of smooth muscle function to preserve blood flow in hypoxic regions during conditions of metabolic stress [21]. Increased reactivity in response to NO donors under hypoxic conditions has already been shown in the placental vasculature [22], and previous studies demonstrated that tone is increased in the human umbilical artery in the presence of high oxygen tension [23].

With regards to the plasma levels of nitrate and nitrite in the fetal circulation, we found no significant difference between normal and FGR pregnancies. Other groups have reported reduced concentrations of nitrite/nitrate in the maternal plasma of FGR pregnancies [12]. Alternatively, higher levels of nitrite have been found in umbilical vein blood samples from FGR pregnancies [24], with some studies even correlating increased nitrite levels with the severity of FGR [25]. The reasons for such between-study discrepancies remain unclear. However, it is worth noting that in these previous studies, reported nitrite concentrations were markedly higher (~30 µmol/L) compared with the values reported in the present study (~1 µmol/L). These previous studies utilized the Griess method to quantify plasma nitrate/nitrite concentrations which, due to its reliance on absorption at 548 nm, may inadvertently measure alternative plasma metabolites that have similar absorption frequencies.

In conclusion, we have demonstrated that enhanced nitrite-mediated vasorelaxation of human placental blood vessels under hypoxic conditions is preserved in CPVs from FGR pregnancies. In addition, we found no differences in the concentrations of nitrate or nitrite within the fetal umbilical vein. Together, these data suggest that there are no overt differences in placental nitrite metabolism in FGR; an important finding, as had deficiencies in nitrite responsiveness been observed in FGR, this would preclude the use of therapeutics targeting this pathway. The attenuated response to SNP in CPVs from FGR pregnancies warrants further investigation, as does the differential responsiveness of CPAs and CPVs to the vasoconstrictor U46619 in FGR compared with normal pregnancies.

From a translational perspective, interventions that increase circulating nitrite concentrations, such as dietary nitrate supplementation, may provide a reservoir of NO-generating potential within the placenta [26]. We have previously shown that beetroot juice supplementation is able to increase plasma nitrate and nitrite concentrations in pregnancy, both in mice and in women [27,28]. Whether such interventions can influence intraplacental vascular function and improve pregnancy outcomes in FGR, or other pregnancy complications associated with raised vascular resistance, remains to be determined, and the molecular mechanisms underlying nitrite actions in the placental vascular bed deserve further investigation.

## 4. Materials and Methods

### 4.1. Samples

A total of 79 placentas were obtained after normal (*n* = 57; no evidence of hypertension, FGR, diabetes or other medical disorders) and FGR pregnancies (*n* = 22, defined according to Delphi consensus [29]); following either elective caesarean section or normal vaginal delivery. Demographic and clinical details of placental donors are summarized in Table 1. Individualized birth weight centiles (IBCs) were calculated using the GROW Centile Calculator (v5.7.7.1, Gestation Network, Perinatal Institute, Birmingham; www.gestation.net accessed on 22 April 2016), which adjusts for: birth weight (g), parity at booking, maternal height (cm), booking weight (kg), ethnic origin, gestation (weeks/days) and baby gender. Placental biopsies were collected into ice-cold physiologic salt solution (PSS; in mM, 117 NaCl, 25 NaHCO3, 4.69 KCl, 2.4 MgSO4, 1.6 CaCl2, 1.18 KH2PO4, 6.05 glucose, 0.034 EDTA; pH 7.4) within 30 min of delivery.

### 4.2. Myography

To perform functional studies, CPAs and CPVs of approximate resistance artery size (diameter < 500 µm) were identified macroscopically from the point of umbilical cord insertion as branches of the umbilical arteries and vein, as previously described [16]. Briefly, chorionic plate vessels were dissected free from the surrounding connective tissues and cut into ~2 mm lengths. After mounting on two 40 μm steel wires in a myograph chamber (Model 620 M, Danish MyoTechnologies, Hinnerup, Denmark), the vessels were immersed in 6 mL of PSS maintained at 37 °C, gassed at two different oxygen tensions (see below) and normalized to an internal diameter of 0.9 L 5.1 kPa, in accordance with Mulvany’s normalization procedure [30].

Following the normalization process, CPAs and CPVs were equilibrated for 20 min prior to the commencement of vasoactive studies. Vessels with diameters > 500 µm were excluded from studies.

To mimic normal conditions of oxygen tension (normoxia) of the placenta, experiments were performed in vessels normalized and equilibrated in 5% CO_2_/5% oxygen/90% nitrogen. Hypoxia was induced by reducing the oxygen tension of PSS with 5% CO_2_/95% nitrogen, as previously described [16].

### 4.3. Functional Experiments

Following equilibration, vessels were exposed to high potassium PSS (KPSS; 120 mM KCl in PSS, equimolar substitution of KCl for NaCl) to assess functional viability. Vessels that were unresponsive to KPSS (generating < 0.1 mN/mm tension following KPSS application) were excluded from the study. After washing twice with PSS, a dose-response curve to the thromboxane mimetic U46619 (10^−10^–2 × 10^−6^ M) was obtained and was used to calculate the EC_80_ concentration of U46619. Steady-state, submaximal pre-constrictions with EC_80_ U46619 were obtained, and either sodium nitrite (NaNO_2_) or the NO donor, sodium nitroprusside (SNP), was then applied to the organ bath in a cumulative fashion (10^−6^–5 × 10^−3^ M and 10^−10^–10^−5^ M, NaNO_2_ and SNP, respectively, at 2–5 min intervals).

### 4.4. Measurement of Nitrate and Nitrite Concentrations in Fetal Plasma

Blood samples were obtained from the umbilical vein and collected into collection tubes (BD 364938, Fisher Scientific, Loughborough, UK) with EDTA added to a final concentration of 2 mM. Immediately, blood was centrifuged, plasma removed and stored at −80 °C until analysis. The relative concentrations of nitrate and nitrite were measured using a high-performance liquid chromatography (HPLC) system (ENO-20; Eicom, Kyoto, Japan), as previously described [31].

### 4.5. Drugs and Chemicals

Chemicals used in this study were purchased from Sigma Aldrich, Poole, UK, unless otherwise stated. U46619 (Merck Chemicals, Gillingham, UK) was applied to the organ bath from frozen small aliquots of stock solutions; NaNO_2_ and SNP stock solutions were prepared daily. All drugs were kept on ice in lightproof vials and further diluted in PSS as required.

### 4.6. Data Analysis and Statistics

Concentration-responses to NaNO_2_ and SNP are expressed as level of constriction remaining from the pre-constriction achieved with an EC_80_ dose of U46619. Maximum relaxation (Vmax) to NaNO_2_ and SNP are expressed as the maximum effect of the dose-response (% relaxation). Effects on the sensitivity (the molar concentration of drugs causing 50% of the maximal response) are expressed as logarithm (LogEC_50_). All data are expressed as mean ± SEM and differences between groups analyzed by two-way ANOVA followed by Tukey’s post hoc test where appropriate. Statistical significance was defined as *p* < 0.05.

## Figures and Tables

**Table 1:** Demographic details of placenta donors.

Demographics		NORMAL FGR Median (IQR)/Number (%)	
Number of Placentas		57	22
Delivery type	C/S	57 (100%)	20 (90.9%)
	NVD	-	2 (9.1%)
Maternal age, years		33 (30–35)	30 (26–36)
Prepregnancy maternal BMI, kg/m^2^		23.94 (21.67–26.99)	24.19 (22.57–26.99)
Maternal smoking		4 (7.0%)	5 (22.7%)
Maternal ethnicity	White/Caucasian	38 (66.7%)	16 (72.7%)
	Asian	12 (21.0%)	3 (13.6%)
	Black	4 (7.0%)	1 (4.6%)
	Other	3 (5.3%)	2 (9.1%)
Gestational age, days ****		273 (267–274)	257 (229–260)
Birth weight, g ****		3232 (2910–3530)	1730 (1261–2317)
Sex: number female (%)		30 (52.6%)	15 (68.2%)
IBC, centile ****		43.30 (26.55–61.80)	0.65 (0.08–1.90)

Data are shown as median and interquartile range (IQR) or as number and percentage as appropriate. Abbreviations: C/S, caesarean section; NVD, normal vaginal delivery; BMI, body mass index; IBC, individualized birthweight centile. **** *p* < 0.0001 normal vs. FGR.

## Data Availability

The primary data presented in this study are available on request from the corresponding authors.

## Supplementary Materials

The following are available online at https://www.mdpi.com/article/10.3390/ijms22094500/s1, Figure S1: U46619-induced vasoconstriction of CPAs and CPVs isolated from normal and FGR placentas, Table S1: Vasorelaxation to NaNO_2_ and SNP in human placental vessels isolated from normal and FGR placentas, Table S2: U46619 EC_80_ doses applied to chorionic plate vessels isolated from normal and FGR placentas.
