# Supplementary material for: Enhanced Nitrite-Mediated Relaxation of Placental Blood Vessels Exposed to Hypoxia Is Preserved in Pregnancies Complicated by Fetal Growth Restriction
Source: Int J Mol Sci. 2021 Apr 26;22(9):4500. doi: 10.3390/ijms22094500 (PMC8123398; doi:10.3390/ijms22094500)
Supplement: Supplementary file 1 [file ijms-22-04500-s001.zip › ijms-1172488-supplementary.pdf]

Supplementary data

Supplementary Table 1

Vasorelaxation to $NaNO_2$ and SNP in human placental vessels isolated from normal and FGR placentas.

Experimental condition		Normal		FGR	
$NaNO_2$	**$LogEC_{50}$ (mol/L)**	**Normoxia**	**Hypoxia**	**Normoxia**	**Hypoxia**
	CPAs	-3.728 ± 0.123	-3.777 ± 0.137	-3.767 ± 0.133	-3.865 ± 0.174
	CPVs	-3.677 ± 0.122	-3.665 ± 0.137	-3.593 ± 0.175	-3.762 ± 0.201
	**Vmax (%)**	**Normoxia[e]**	**Hypoxia**	**Normoxia[e]**	**Hypoxia**
	CPAs[c]	59.7 ± 4.8 [b]	69.7 ± 4.2 [b]	58.4 ± 6.5	73.0 ± 4.1 [b]
	CPVs	40.4 ± 4.0	53.0 ± 4.5	40.3 ± 5.3	46.5 ± 3.7
SNP	**$LogEC_{50}$ (mol/L)**	**Normoxia [b]**	**Hypoxia**	**Normoxia [b]**	**Hypoxia**
	CPAs [a]	-7.624 ± 0.186	-7.226 ± 0.191	-7.302 ± 0.241	-7.251 ± 0.197
	CPVs [g]	-7.176 ± 0.147	-7.385 ± 0.134	-6.611 ± 0.183	-6.869 ± 0.186
	**Vmax (%)**	**Normoxia [f]**	**Hypoxia**	**Normoxia[e]**	**Hypoxia**
	CPAs	66.6 ± 5.2	75.8 ± 3.6	68.1 ± 6.6	79.4 ± 5.3
	CPVs	61.7 ± 4.3 [f]	79.8 ± 3.3	57.1 ± 4.6	73.6 ± 5.1

$LogEC_{50}$ (logarithm of the molar concentration of each drug causing 50% of the maximal relaxation), and Vmax (maximum relaxation), for $NaNO_2$- and SNP-mediated vasorelaxation in each experimental condition (see Results in the main text). [a] $p < 0.05$, [b] $p < 0.01$, [c] $p < 0.001$, [d] $p < 0.0001$, CPAs vs CPVs; [e] $p < 0.05$, [f] $p < 0.01$ normoxia vs hypoxia; [g] $p < 0.05$, Normal vs FGR. All data are mean ± SEM.

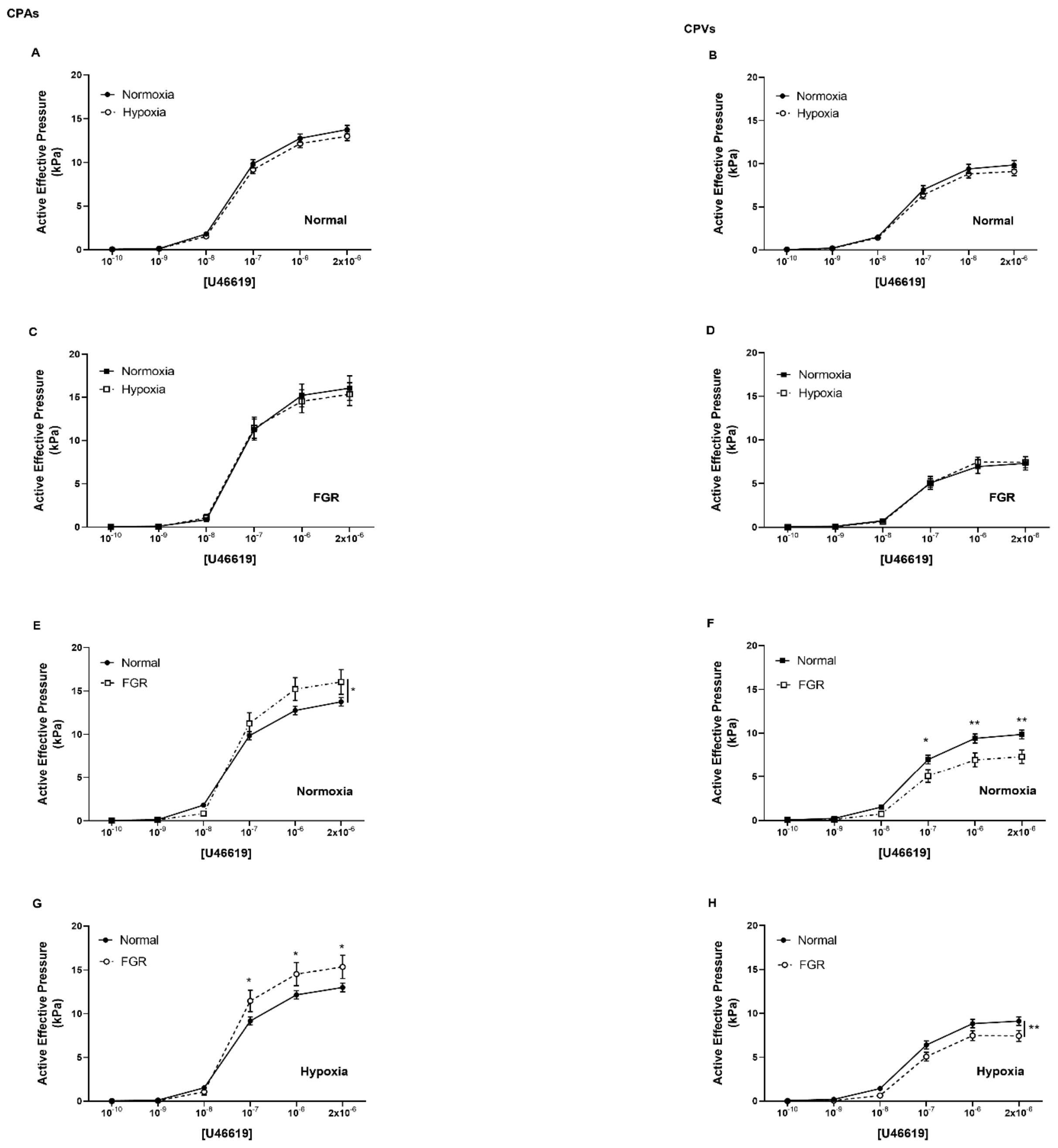

Supplementary Figure 1. U46619-induced vasoconstriction of CPAs and CPVs isolated from normal and FGR placentas. Concentration-dependent vasoconstricting effect of U46619 on: normal CPAs (A) and CPVs (B), FGR CPAs (C) and CPVs (D), in normoxia and hypoxia; normoxic CPAs (E) and CPVs (F), hypoxic CPAs (G) and CPVs (H), from normal and FGR pregnancies. * $p < 0.05$, ** $p < 0.01$, normal vs FGR. Data are presented as active effective pressure, expressed in kPa. All data are mean ± SEM. n = 16-75 placentas.

Supplementary Table 2

U46619 EC_{80} doses applied to chorionic plate vessels isolated from normal and FGR placentas.

**U46619 $LogEC_{80}$ (mol/L)**	**Normal**		**FGR**	
Vessel types	Normoxia [a]	Hypoxia [a]	Normoxia [b]	Hypoxia [b]
CPAs	-6.945 ± 0.016 [a]	-6.930 ± 0.0158	-6.981 ± 0.019 [a]	-6.963 ± 0.020 [a]
CPVs	-6.867 ± 0.030	-6.901 ± 0.030	-6.829 ± 0.058	-6.803 ± 0.064

$LogEC_{80}$, logarithm of the molar concentration of U46619 causing 80% of the maximal vasoconstriction prior to assessment of dose-response curves to either $NaNO_2$ or SNP. CPVs were significantly less sensitive to U46619 and required a higher concentration of the thromboxane mimetic to achieve EC_{80} pre-constriction compared with CPAs ([a] $p < 0.05$, [b] $p < 0.01$, CPAs vs CPVs). Oxygen tension did not affect U46619 EC_{80} concentrations in either vessel types. All data are mean ± SEM. n = 16-75 placentas.
